# Chlorophyll Catabolites – Chemical and Structural Footprints of a Fascinating Biological Phenomenon

**DOI:** 10.1002/ejoc.200800804

**Published:** 2008-12-02

**Authors:** Simone Moser, Thomas Müller, Michael Oberhuber, Bernhard Kräutler

**Affiliations:** [a] Institute of Organic Chemistry and Centre of Molecular Biosciences, University of InnsbruckInnrain 52a, 6020 Innsbruck, Austria, Fax: +43-512-507-2892 E-mail: bernhard.kraeutler@uibk.ac.at

**Keywords:** Natural products, Chlorophyll, Catabolism, Biomimetic synthesis, Structure elucidation, Tetrapyrroles

## Abstract

Twenty years ago, the molecular basis for the seasonal disappearance of chlorophyll was still enigmatic. In the meantime, our knowledge on chlorophyll breakdown has grown considerably. As outlined here, it has been possible to decipher the basic transformations involved in natural chlorophyll breakdown by identification of chlorophyll catabolites in higher plants, and with the help of the synthesis of (putative) catabolic intermediates. In vascular plants, chlorophyll breakdown typically converts the green plant pigments efficiently into colorless and non-fluorescent tetrapyrroles. It involves colored intermediates only fleetingly and in an (elusive) enzyme-bound form. The non-fluorescent chlorophyll catabolites accumulate in the vacuoles of degreened leaves and are considered the products, primarily, of a detoxification process. However, they are effective antioxidants, and may thus also have physiologically beneficial chemical properties.(© Wiley-VCH Verlag GmbH & Co. KGaA, 69451 Weinheim, Germany, 2009)

## Introduction

The seasonal metabolism of the chlorophylls is probably the most visual sign of life on earth, observable even from outer space.^1^ It is estimated that more than 10^9^ tons of chlorophyll (Chl) are biosynthesized and degraded every year on the earth.^2^ The appearance of the green plant pigments in spring and their disappearance in the autumnal foliage of deciduous trees and in ripening fruit belong to the most colourful and fascinating natural phenomena. However, only within the last two decades has Chl breakdown in plants begun to yield some of its mysteries.^1^,^3^–^6^

This chapter reviews our knowledge on the occurrence, structures and reactivities of chlorophyll catabolites from vascular plants, and recapitulates today's synthetic advances for preparing chlorophyll catabolites and related tetrapyrroles. The available structural information provides a basis for deriving much of the current insight into the biochemical pathways of chlorophyll breakdown in higher plants, as has been reviewed elsewhere recently.^4^–^6^

## Structural Identification of Chlorophyll Catabolites from Senescent Leaves

### Colorless and Nonfluorescent Chlorophyll Catabolites

**Scheme 1:**
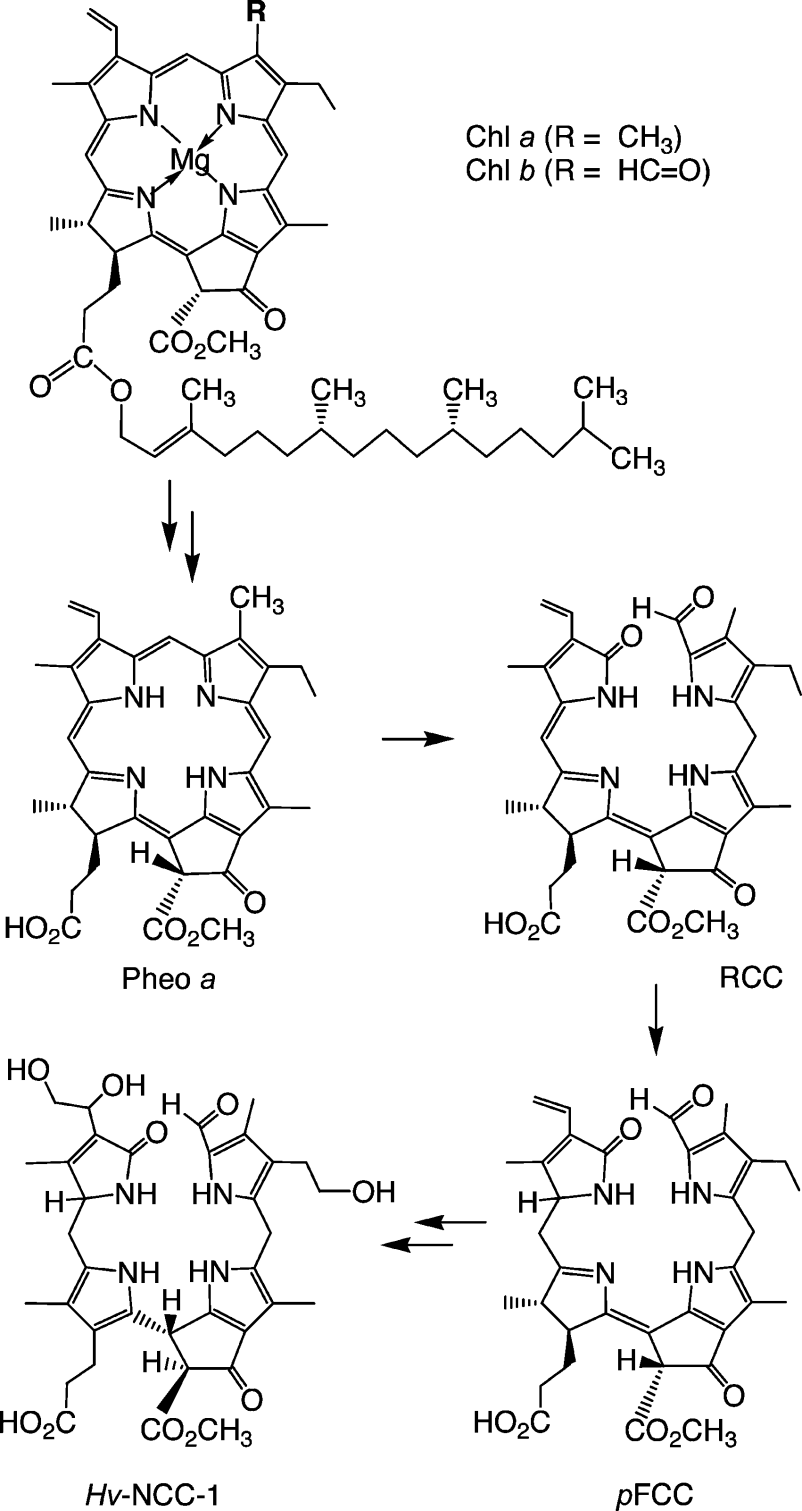
Tetrapyrrolic chlorophyll catabolites in senescent higher plants.^4^,^5^ Chlorophylls [Chl *a* (R = CH_3_) or Chl *b* (R = CH=O)] are degraded via pheophorbide *a* (Pheo *a*), the “red” chlorophyll catabolite (RCC) and primary “fluorescent” chlorophyll catabolites (pFCCs) to “nonfluorescent” chlorophyll catabolites (NCCs, such as *Hv*-NCC-1, **1**).

Matile and co-workers provided the first evidence for the existence – in the vacuoles in senescent leaves of *Festuca pratensis*^7^,^8^ and barley (*Hordeum vulgare*)^9^,^10^ – of colourless Chl catabolites that readily decomposed into rust-coloured compounds.^11^ The main catabolite from barley, now named *Hv*-NCC-1 [**1**, a 3^1^,3^2^,8^2^-trihydroxy-1,4,5,10,15,20-(22*H*,24*H*)-octahydro-13^2^-(methoxycarbonyl)-4,5-dioxo-4,5-*seco*-phytoporphyrinate], was the first colourless Chl catabolite from higher plants to be identified (see Scheme 1).^3^,^12^ The ‘non-fluorescent' catabolite (NCC) **1** was thus revealed to be a tetrapyrrole derived from Chls and to carry a methyl group at the 7-position [i.e., to be (more closely) related to Chl *a* (rather than to Chl *b*)]. Its structure gave the first hints of the transformations of Chls during their breakdown: it is a metal-free, linear tetrapyrrole with deconjugated pyrrole units, reflecting oxygenolytic opening of the macro ring of Chl *a* at the northern *meso* position and loss of the central magnesium ion. In addition, several of the peripheral groups carry functional units different from those in Chl *a* and feature polar groups that make the NCCs more soluble in water.

The initial identification of the colourless, nonfluorescent chlorophyll catabolite *Hv*-NCC-1 was followed by an intensive search in senescent leaves from a variety of vascular plants. Various NCCs were identified, from oilseed rape (*Brassica napus*, four *Bn*-NCCs^13^,^14^), from tobacco (*Nicotiana rustica*, two *Nr*-NCCs^15^), from maize (*Zea mais*, two *Zm*-NCCs^16^) and from degreened leaves of spinach (*Spinaccia oleracea*, five *So*-NCCs^17^,^18^) (see Table 1).

**Table 1:** List of nonfluorescent chlorophyll catabolites (NCCs) from higher plants with a common general formula and varying modifications R^1^, R^2^ and R^3^[a]

	Compound	R^1^	R^2^	R^3^	Ref.	
**1**	*Hv*-NCC-1	OH	CH_3_	CH(OH)–CH_2_OH	3,12	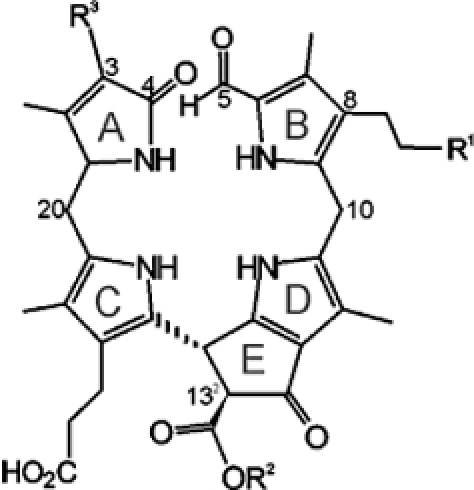
**2**	*Cj*-NCC-1/*So*-NCC-4/*Pc*-NCC-2/*Ms*-NCC-2	OH	CH_3_	CH=CH_2_	[18–20,47]	
**3**	*Cj*-NCC-2/*So*-NCC-5	H	CH_3_	CH=CH_2_	[18–20]	
**4**	*Bn*-NCC-1	O-Mal	H	CH=CH_2_	[13,14]	
**5**	*Bn*-NCC-2/*At*-NCC-1	O-β-(6′-O-Mal)Glc	H	CH=CH_2_	[14,23]	
**6**	*Bn*-NCC-3/*At*-NCC-2	OH	H	CH=CH_2_	[14,23]	
**7**	*Bn*-NCC-4/*At*-NCC-5	H	H	CH=CH_2_	[23]	
**8**	*At*-NCC-4	O-β-Glc	CH_3_	CH=CH_2_	[23]	
**9**	*So*-NCC-1	OH	H	CH(OH)–CH_2_OH	[18]	
**10**	*So*-NCC-2	OH	CH_3_	CH(OH)–CH_2_OH	[17,18]	
**11**	*So*-NCC-3	OH	H	CH=CH_2_	[18]	
**12**	*Nr*-NCC-1	O-β-(6′-O-Mal)Glc	CH_3_	CH=CH_2_	[15]	
**13**	*Nr*-NCC-2/*Zm*-NCC-2 *Pc*-NCC-1	O-β-Glc	CH_3_	CH=CH_2_	[15,16,47]	
**14**	*Zm*-NCC-1	O-β-Glc	CH_3_	CH(OH)–CH_2_OH	[16]	

[a] Abbreviations: Mal = malonyl; Glc = glucopyranosyl.

All NCCs described so far have exhibited the same basic structural pattern, involving four deconjugated pyrrolic units and an α-formyl group at ring B, derived from the former α-*meso* carbon of Chl *a*. A methyl group at the 7-position indicates a close relationship of natural NCCs to Chl *a*.^3^,^5^ A hydroxy group at the terminal 8^2^-position of the ethyl side chain at ring B is another remarkable feature, which increases the polarity of the catabolites and serves as an anchor for further attachment of hydrophilic groups at the 8^2^-position.^3^,^5^ There is additional constitutional variability at the side chains at the 3- and 13^2^-positions, all resulting from peripheral modifications during catabolism (see Table 1). Interestingly, while the major catabolite (*Cj*-NCC-1) from senescent leaves of the Katsura tree (*Cercidiphyllum japonicum*) followed this general pattern,^19^ the less polar catabolite (*Cj*-NCC-2) lacked the puzzling hydroxy function at ring B, so it is an isomer of the *primary* (unmodified version of the) fluorescent chlorophyll catabolite and has also been named as a *primary* NCC (see below).^20^

Nowadays, NCCs can be identified in extracts of senescent leaves on the basis of their characteristic UV/Vis absorption properties: because of the α-formyl pyrrole moiety at ring B, they show prominent absorption maxima near 320 nm. ESI-MS spectra of NCCs help to establish their molecular formulas and to identify the available functional groups. Characteristic fragmentation patterns arise from the loss of rings A and B and allow conclusions to be drawn with regard to the distributions of functional groups at these two rings. Furthermore, the methyl ester function at the 13^2^-position frequently gives rise to a fragment ion lacking a mass of 32; otherwise, decarboxylation can be observed readily for a free acid group at this position.^16^

The constitutions of the NCCs could be delineated by one- and two-dimensional NMR measurements. In the ^1^H spectrum of a typical nonfluorescent chlorophyll catabolite, a CH=O group singlet at low field, the spin system for a vinyl group (when present) at an intermediate field, a methyl ester singlet and four singlets of the four pyrrole-bound methyl groups at high field usually stand out.^3^,^21^

Natural NCCs all have similar CD spectra, suggesting a common absolute (*R*) configuration at the C(15) *meso* position.^20^ In contrast, the NCCs occur in two epimeric classes, due to different absolute configurations at C(1) (see, for example, ref.^18^ and below).

**Figure 1:**
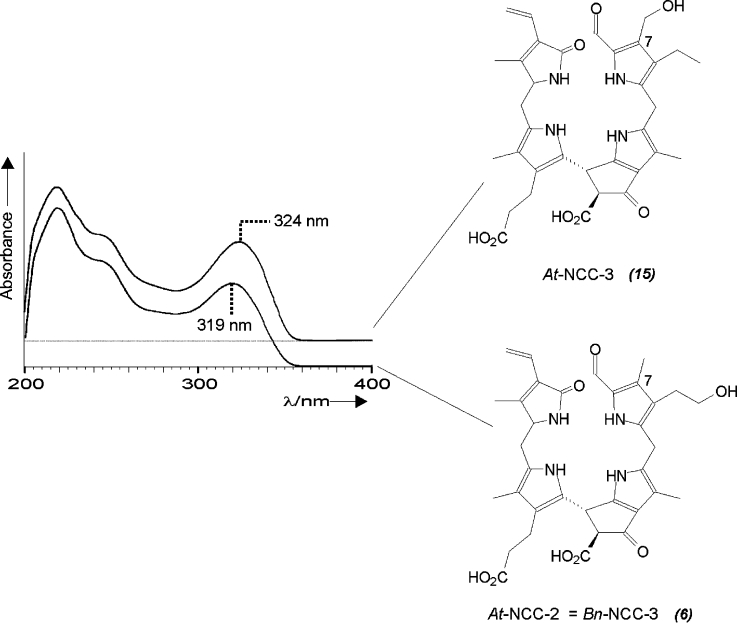
UV absorbance spectra and constitutional formulae of *At*-NCC-3^22^ and *At*-NCC-2 (= *Bn*-NCC-3), an example of a typical natural NCC with a methyl group at C(7).^23^

A first example indicative of a divergent path of chlorophyll breakdown was found in extracts of senescent leaves of the model plant *Arabidopsis thaliana*:^22^ five fractions with the UV/Vis characteristics of NCCs were identified (termed *At*-NCC-1 to *At*-NCC-5) and their constitutions were determined by NMR and MS measurements.^23^ The NMR spectra revealed an interesting new pattern of the substituents for *At*-NCC-3. Only three singlets appeared at high field, and complete assignment of the ^1^H and ^13^C signals from ROESY, HSQC and HMBC spectra revealed the group attached at C(7) to be a hydroxymethyl group.^22^ This type of substituent at the α-formyl pyrrole moiety causes a shift in the absorption maximum in the UV/Vis spectrum to 324 nm (see Figure 1). This unique modification of an NCC from a higher plant at C(7) indicated a modified catabolic pathway in *A. thaliana*, suggesting the occurrence of one of two alternative deviations from the ‘normal’ path. Either i) Chl *b* was not completely reduced and pheophorbide *a* oxygenase (the enzyme that cleaves the chlorin macrocycle; see below) accepts 7-hydroxymethyl pheophorbide *a* as a substrate, or else ii) the (enigmatic) side chain hydroxylation at C(8^2^) is unselective. The introduction of this hydroxy function is believed to occur at the stage of an FCC and is likely to be a two-step radical reaction (i.e., either the radical-forming primary H abstraction is unselective, or incorporation of oxygen takes place other than at the original radical site).^22^

Simone Moser (second from left) was born in Rum (Austria) in 1981. She studied chemistry at the University of Innsbruck. After finishing her diploma thesis on chlorophyll catabolites in Arabidopsis thaliana with Prof. Bernhard Kräutler, she continued her work, on chlorophyll breakdown in bananas, as a Ph.D. student under the guidance of Prof. Bernhard Kräutler in 2004.

Thomas Müller (right) was born in Schwaz (Austria) in 1971. He studied chemistry at the University of Innsbruck. After completing his diploma thesis on fullerenes with Prof. Bernhard Kräutler, he worked as a chemistry teacher in Innsbruck and began his Ph.D. work on capillary electro-chromatography with Prof. Herbert Lindner at the Medical University of Innsbruck, where he obtained his Ph.D. degree in November 2002. Since July 2003 he has been working as an Assistant Professor at the University of Innsbruck in the Kräutler group. Research interests: molecular life sciences, mass spectrometry.

Michael Oberhuber (second from right)was born in 1974 in Bruneck, Italy. He obtained a diploma in chemistry and received a Ph.D. degree in organic chemistry at the University of Innsbruck, Austria. In 2002 he completed his Ph.D. studies under the supervision of Prof. B. Kräutler on the topic of a biomimetic partial synthesis of chlorophyll catabolites. He then joined the laboratory of Prof. G. F. Joyce at The Scripps Research Institute, La Jolla, CA, where he studied DNA-templated chemistry and directed evolution of RNA enzymes. After returning to the University of Innsbruck as an Assistant Professor in the Kräutler group in 2005, he was offered a position and joined the Sandoz Development Center at Sandoz GmbH, Kundl, Austria in 2008. Research interests: bioorganic chemistry, nucleic-acid-templated chemistry, and molecular evolution.

Bernhard Kräutler (left) was born in Dornbirn (Austria) in 1946. He studied chemistry at the ETH in Zürich, where he received his Ph.D. in 1976 under the guidance of Prof. Albert Eschenmoser. Following his postdoctoral studies with Prof. Allen J. Bard (University of Texas at Austin) and Prof. Nicholas J. Turro (Columbia University, New York) he returned to the ETH. After his habilitation in 1985 at the ETH, he was Visiting Professor, University of Illinois in Urbana (fall 1985) and was called in 1991 as Full Professor of Organic Chemistry to the University of Innsbruck, where he is currently the head of the Institute of Organic Chemistry. Interests in molecular life sciences and molecular engineering; current research topics: chlorophyll breakdown in leaves and fruit; chemical biology of vitamin B_12_ and B_12_-binding macromolecules; functionalized fullerenes and porphyrins.

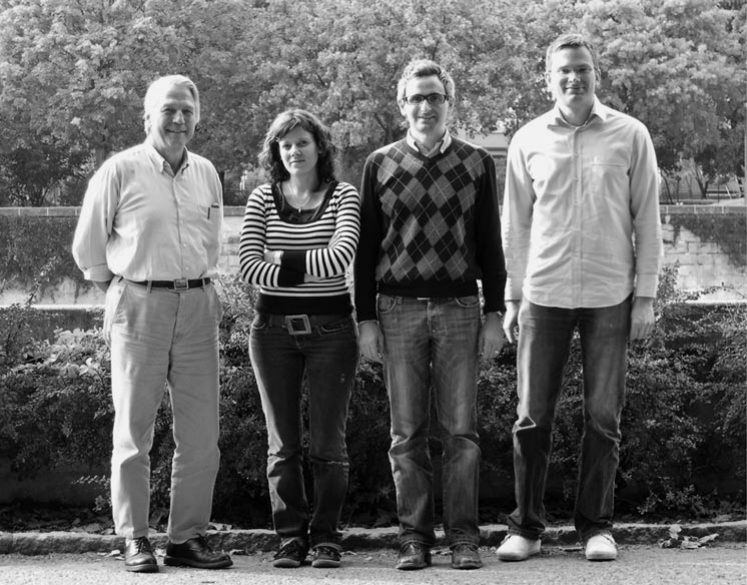

### Colorless and Fluorescent Chlorophyll Catabolites

The direct precursors of the NCCs have been identified as ‘fluorescent' chlorophyll catabolites (FCCs).^24^ Under most circumstances, however, FCCs are only fleetingly observed intermediates of chlorophyll breakdown. Typical natural FCCs easily undergo chemical isomerization under slightly acidic conditions and convert into NCCs (as also described in the next section).

**Scheme 2:**
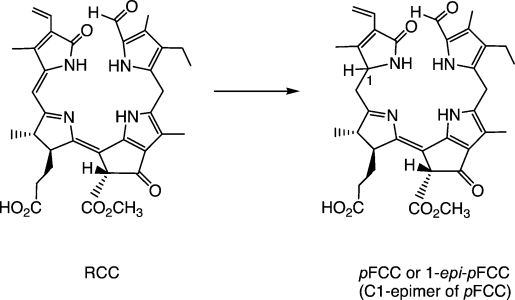
Two stereodivergent lines of RCC reductases occur in (senescent) higher plants, each giving one of two epimeric “primary” FCCs (*p*FCCs) by stereoselective reduction of (enzyme-bound) red chlorophyll catabolite (RCC).^26^,^27^

Minute amounts of FCCs were detected early, due to their fluorescence (they show emission with a maximum at 450 nm), and FCCs were suggested as intermediate chlorophyll breakdown products.^25^ It was possible to produce a small quantity of a naturally occurring FCC from the transformation of pheophorbide *a* (Pheo *a*) by an enzymatically active extract of senescent leaves of *B. napus* (oilseed rape) and to elucidate its structure.^24^ Its UV/Vis spectrum differed from that of an NCC in an additional absorption maximum at 360 nm, due to a chromophore extending over rings C and D.^24^ Its molecular formula confirmed it to be a chlorophyll catabolite, derived from Pheo *a* by addition offour hydrogen and two oxygen atoms. Our analysis led us to suggest the existence of an elusive red-coloured precursor, now called red chlorophyll catabolite (RCC), enzyme-catalysed reduction of which would furnish this FCC directly (see below), which was therefore named the ‘primary' fluorescent chlorophyll catabolite (*p*FCC).^24^ Another FCC was obtained from related experiments with extracts of senescent leaves of sweet pepper (*Capsicum annuum*); it turned out to be the C(1) epimer of pFCC and was thus named *epi*-pFCC.^26^ As the configurations of the C(1) stereocentres of the FCCs were assumed to be determined in reductions catalysed by RCC-reductase,^27^ the occurrence of two lines of stereoselective RCC reductases in the higher plants was indicated, that produce either *p*FCC or *epi-p*FCC (see Scheme 2).^28^

## Chlorophyll Catabolites and Related Tetrapyrroles from Partial Synthesis

Chemical synthesis of presumed earlier products of chlorophyll breakdown became a target, as Chl breakdown was found typically to proceed to NCCs rapidly, without producing significant amounts of intermediates. On one hand, our synthetic work was thus motivated as a means of preparing postulated, but elusive, earlier products of chlorophyll catabolism (such as the red catabolite, RCC). On the other hand, it also provided (intermediate) catabolites that were scarce in natural sources (such as the FCCs). The synthetic approach was also an impetus for reconstructing chlorophyll catabolism through biomimetic conversions, as a means of testing enzyme mechanistic aspects of chlorophyll breakdown. In addition, it was to yield appreciable amounts of potential intermediates for further studies.

Green intermediates of chlorophyll breakdown, such as Pheo *a*, were known to be available from Chl *a*. Loss of the central Mg^2+^ ion, for instance, occurs spontaneously in dilute acid. Indeed, intact Chls are not isolated from typical preparative procedures. Instead, Pheo esters – such as Pheo *a* methyl ester – are conveniently obtained as crystalline solids.^29^,^30^

### Preparation of the Elusive Red Chlorophyll Catabolite (RCC) by Partial Synthesis

The crucial step in natural Chl breakdown was for a long time believed to be an oxygenolytic opening of the chlorin ring.^[2,4,24,31,32]^ As a synthetic version of this, cleavage of the chlorin ring of Pheo *a* in a photooxidation reaction could be envisaged (this would probably involve singlet oxygen and a formal [2+2] cycloaddition reaction). However, photochemical studies of (metallo-)chlorins^32^ and their known reactivity towards electrophilic reagents^33^ suggested the western *meso* position as the most susceptible for oxygenolysis.^32^ The first structures of marine natural products derived from Chls^34^,^35^ appeared to strengthen the significance of his cleavage pattern. In obvious contrast, NCCs were accessible from Chls by cleavage at the northern *meso* position.^3^,^24^,^31^ Indeed, this oxygenolytic cleavage of the porphyrinoid macroring is achieved at the level of Pheo *a* by a mono-oxygenase, now called Pheo *a* oxygenase (PaO).^31^

**Scheme 3:**
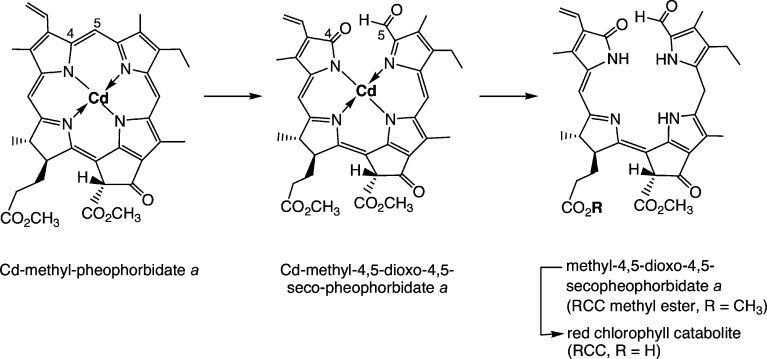
Outline of partial synthesis of red chlorophyll catabolite (RCC) and its methyl ester (Me-RCC).^37^

Experiments in Gossauer's lab revealed Cd^2+^ complexes of pyropheophorbide *a* to be good starting materials for obtaining the desired [4,5]-*seco*-[4,5]-dioxo regioisomer as a main oxygenation product.^36^ When the Cd^2+^ complex of Pheo *a* methyl ester^29^,^30^ was photooxidized analogously, the corresponding [4,5]-*seco*-[4,5]-dioxo regioisomer was obtained in about 35 % yield (see Scheme 3),^37^ together with the corresponding [19,20]-*seco*-[19,20]-dioxo isomer as a minor product (10 %).^38^ The brownish and rather unstable oxidation product was readily reduced with NaBH_4_ to give a deep-red compound, which was identified as the methyl ester of RCC by spectroscopic analysis.^37^ Its enzymatic hydrolysis with porcine liver esterase selectively involved the less hindered (propionic acid) ester, giving the first sample of authentic RCC in nearly quantitative yield.^37^ This compound proved to be a substrate for further conversion into an FCC in the presence of extracts from senescent rape cotyledons, identified with *p*FCC in an HPLC assay.^39^,^40^ With synthetic RCC available as reference material, traces of the very same (elusive) red compound were detected when Pheo *a* was incubated with extracts of senescent chloroplasts.^39^,^40^

### Electrochemical Reduction of the Red Chlorophyll Catabolite (RCC) to FCCs

**Scheme 4:**
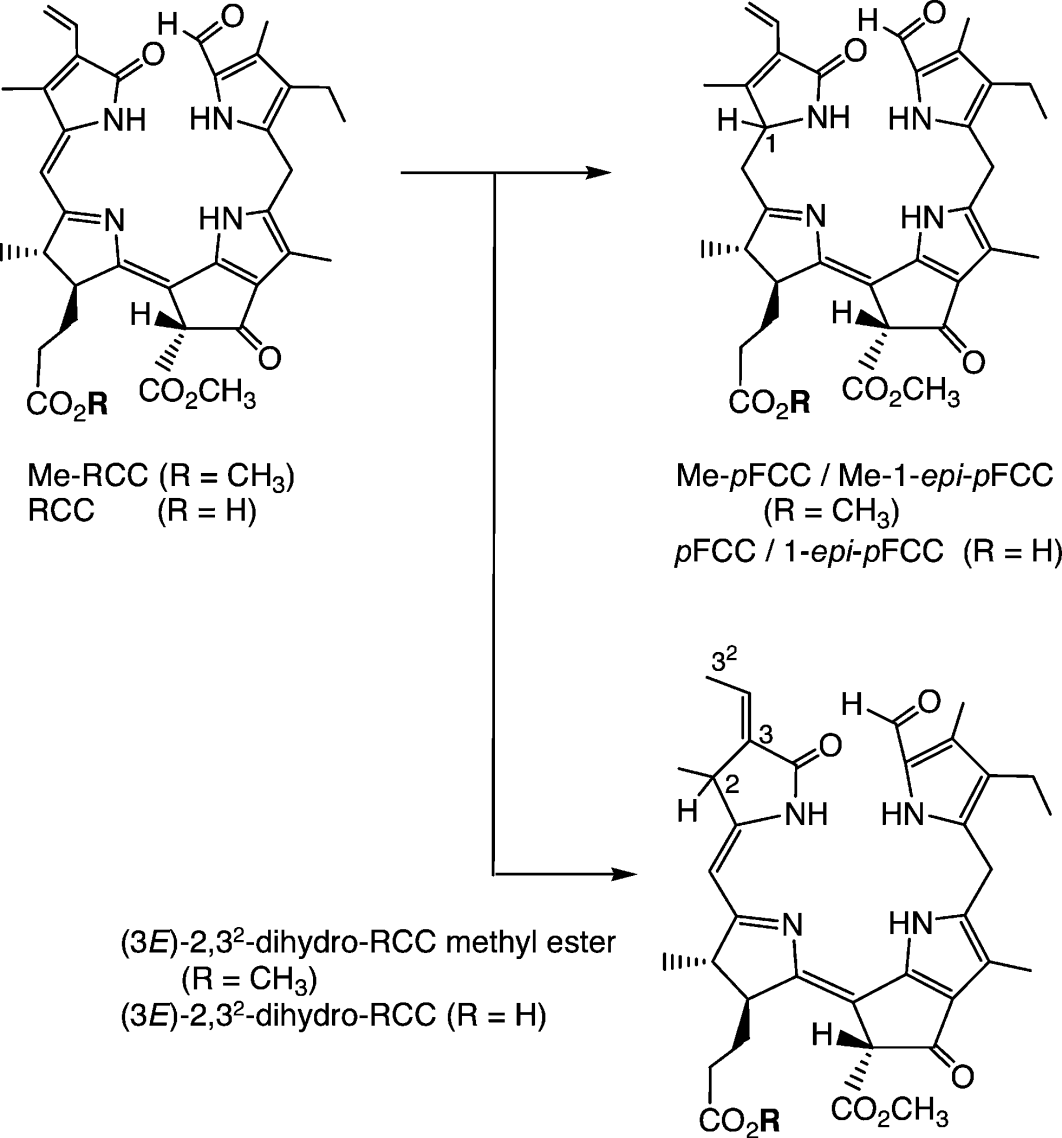
Electrochemical reduction of red chlorophyll catabolite (RCC) or of its methyl ester (Me-RCC) leads to the two epimeric primary FCCs (*p*FCC and *epi-p*FCC) or their methyl esters, respectively, as well as to regioisomeric 2,3^2^-dihydro-RCCs and their methyl esters.^41^,^42^

In senescent leaves, RCC is stereoselectively converted into the “primary” FCCs, mediated by the action of two types of ferredoxin-dependent RCC reductases.^28^ This biochemical finding was paralleled in a biomimetic reaction involving the electrochemical reduction of RCC methyl ester, or RCC itself, yielding four fluorescent products (≈ 25 %) and two major yellow isomers (≈ 30 %) in both types of experiments (see Scheme 4).^41^,^42^ The fluorescent fractions were shown to represent all possible stereoisomers of FCCs (or their methyl esters), revealing that the electrochemical reduction proceeded with acceptable regioselectivity at the 1- and 20-positions, but lacked significant stereoselectivity.^41^,^42^ This experiment thus gave both distinct C(1)-epimeric lines of FCCs, which are also formed in higher plants (depending on the class of reductase present).^24^,^26^,^27^ The yellow fractions were isomeric products, in which reduction had occurred at a different position.^41^,^42^ These latter regioisomers (2,3^2^-dihydro-RCCs) were reminiscent of the structures of some phycobilins, plant pigments derived from biliverdin by the action of related biliverdin reductases.^43^ From the facile electrochemical reduction of RCCs to FCCs, RCCs appear to be sufficiently redox-active to undergo ferredoxin-driven reduction to FCCs. The high regio- and stereoselectivities of protonation steps, which alternate with single-electron reductions in the course of the overreduction of RCC to an FCC, would thus remain as the apparent main task of (the enzyme) RCC reductase.^41^,^42^

### Isomerization of FCCs to NCCs

**Scheme 5:**
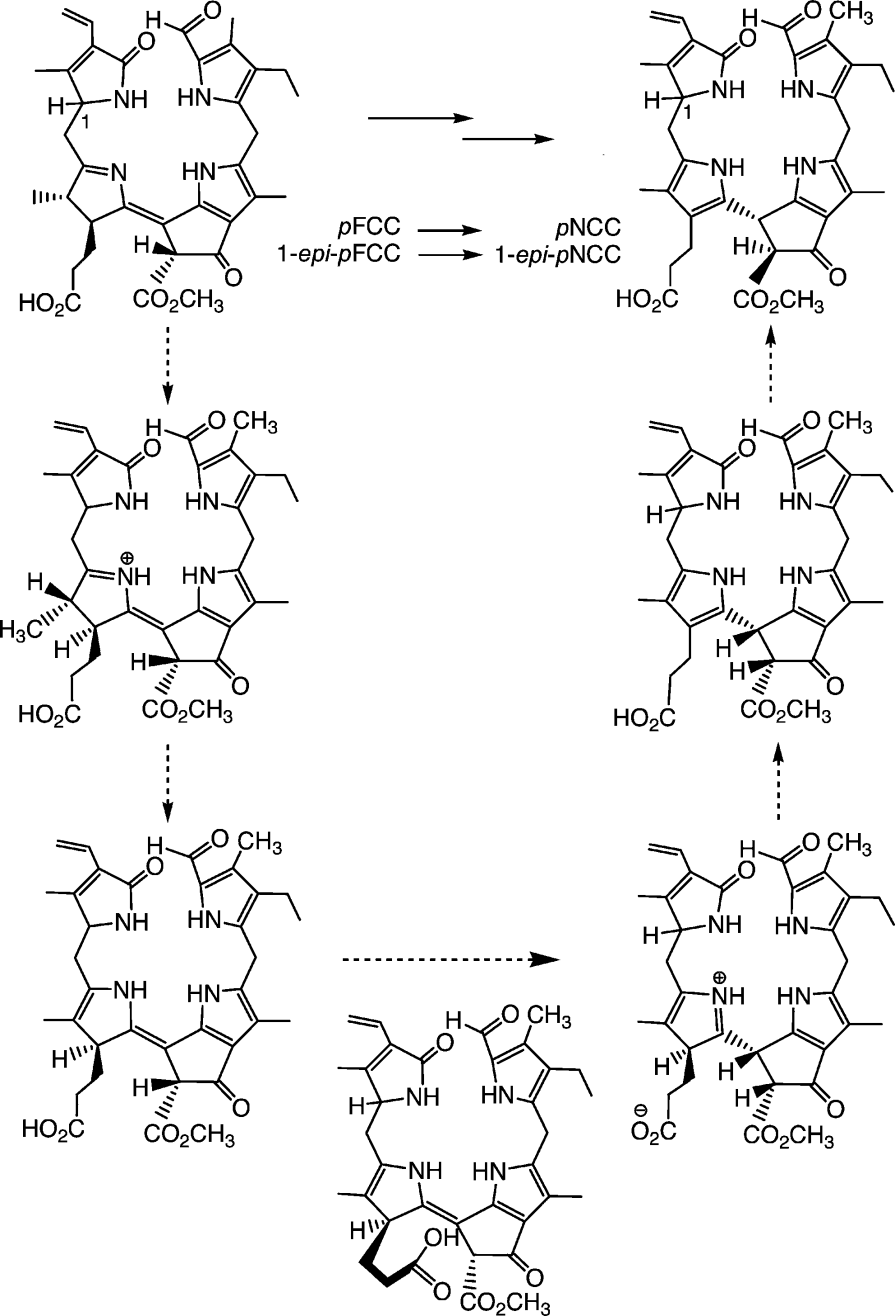
Isomerization of the epimeric “primary” FCCs (*p*FCC and *epi-p*FCC) is catalysed stereoselectively by the propionic acid function and leads to “primary” NCCs (*p*NCC and *epi-p*NCC), as interpreted in terms of the suggested mechanism shown.^20^,^42^

The structural relationship between FCCs and NCCs suggested that FCCs could be convertible into NCCs through nonenzymatic isomerization, with rearomatization of ring D as a thermodynamic driving force.^44^ Such a reaction would occur at the expense of deconjugation of rings C and D, yielding the four isolated pyrrole units characteristic of the NCC chromophore. This hypothesis was tested by exposing *epi-p*FCC (obtained from Pheo *a* by treatment with an enzyme extract of *Capsicum annuum*)^26^ to slightly acidic conditions under inert gas.^20^ Analysis of this reaction mixture by HPLC after 18 h revealed that the FCC had been completely converted into an NCC, identified as the natural *Cj*-NCC-2 (or *epi-p*NCC) from the Katsura tree. A remarkable detail of this reaction was the appearance of an NCC intermediate (with *t*_1/2_ ca. 100 min), which slowly converted into *Cj*-NCC-2 (with *t*_1/2_ ca. 600 min). From the well known epimerization of β-keto esters, the intermediate NCC was tentatively identified as the C13^2^-epimer of *Cj*-NCC-2. This experiment provided good evidence that i) FCCs isomerize quickly to NCCs when they are exposed to acidic conditions and that ii) protonation occurs highly stereoselectively, giving uniform configuration at C(15).^20^ This reaction was also investigated with the epimeric *p*FCC from synthetic sources (vide supra), which was efficiently converted to *p*NCC as major product. Tautomerization occurred with apparent first-order rate constants of 0.02 (*p*FCC) and 0.039 min^−1^ (*epi*-*p*FCC), respectively, at pH 4.0. A pH profile of the reaction rate was consistent with the participation of a proton donor with a p*K*_a_ near 5,^42^ suggesting that the isomerization was achieved through an intramolecular protonation of C15 by the propionic acid side chain extending from ring D. This intramolecular protonation could occur in a stereospecific manner from the β-side only, giving the (*R*) configuration at C(15) (see Scheme 5).^20^ This first isomerization would be followed by an epimerization at C(13^2^), yielding *Cj*-NCC-2 (*epi-p*NCC), with its suggested (15*R*,13^2^*S*) configuration. From these studies, we proposed that this last step of chlorophyll breakdown should occur non-enzymatically in the acidic milieu of the vacuoles, in which the free propionic acid function would be largely protonated. The intramolecular protonation would result uniformly in (*R*) configurations at C15 in all natural NCCs, as is consistent with their very similar CD spectra. The chemical isomerization of *p*FCC/*epi*-pFCC to *p*NCC/*epi-p*NCC also completed the partial synthesis of natural NCCs from the starting Chl *a*.^42^

**Figure 2:**
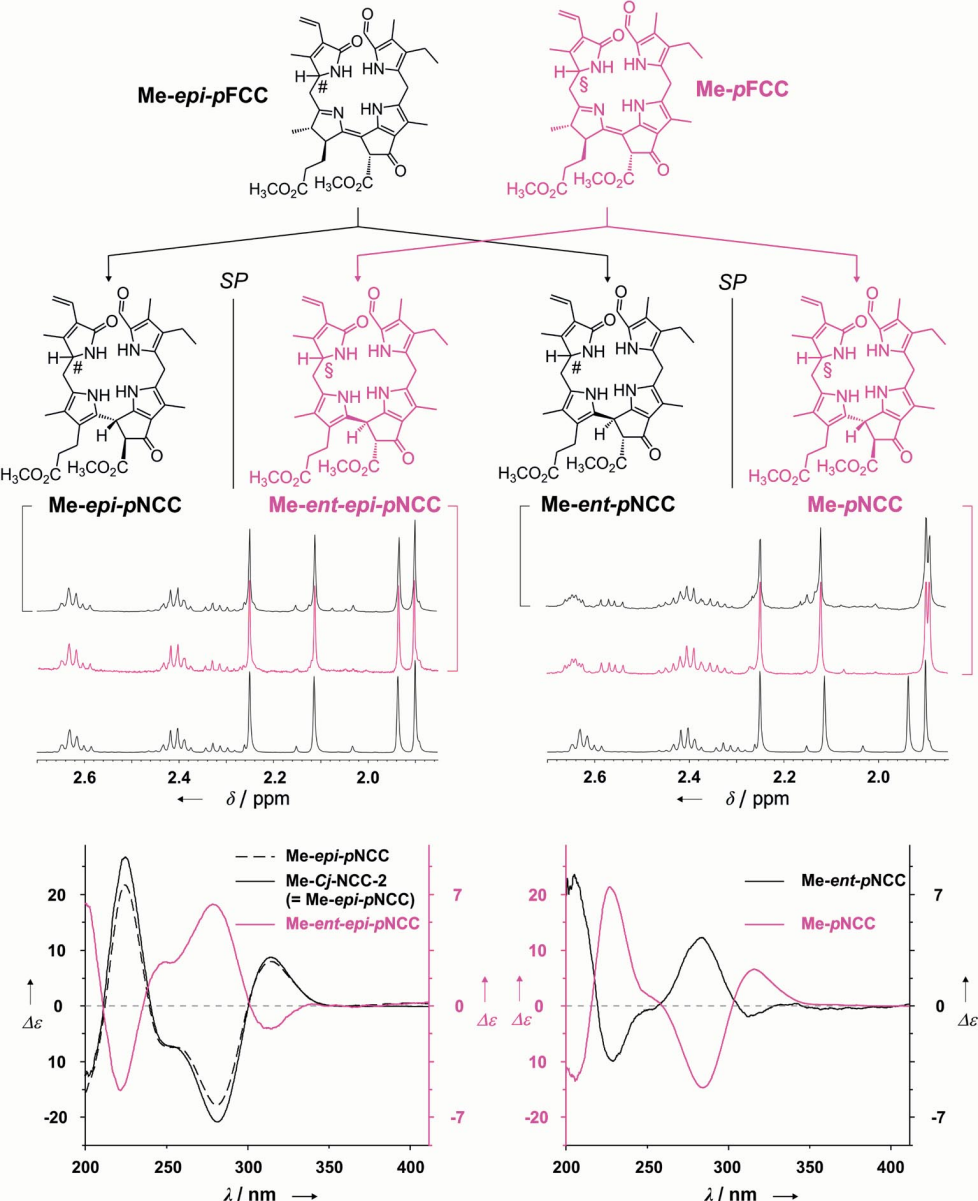
Isomerization of “primary” FCC methyl esters occurs with little stereoselectivity and gives NCC methyl esters of the “primary” NCCs as well as their enantiomers.^42^

The critical role of the propionic acid side chain in the FCC-to-NCC isomerization was further demonstrated by study of this type of isomerization reaction with the related FCC methyl esters. Both epimeric lines (Me-*p*FCC and Me-*epi-p*FCC) isomerized with low stereoselectivities, and only at much slower rates, when exposed to more acidic conditions [giving mixtures of C(15) stereoisomers of NCC methyl esters; see Figure 2].^42^ On the one hand, these results confirmed the involvement of the propionic acid functionality both in the high reaction rate and in the stereospecificity of the FCC tautomerization. On the other, the stereochemically unselective version involving the FCC methyl esters provided unprecedented preparative access to epimeric NCCs with the (*S*) configuration at C(15), and thus enantiomers of some natural NCCs. Indeed, the CD spectra of the minor stereoisomers isolated from the reaction mixture were nearly mirror images of their natural counterparts, while their NMR spectra were identical. The conversion of NCC methyl esters into NCCs was accomplished through enzymatic hydrolysis involving porcine liver esterase, also concluding the formal partial synthesis of the enantiomers of natural NCCs.^42^ Surprisingly, accumulation of natural FCCs was recently observed in ripening banana in our laboratory and could be linked to esterification of the propionic acid in these intermediary catabolites, pointing to physiological relevance for natural esters of FCCs (see below^48^).

## Structural Identification of Chlorophyll Catabolites from Fruit

It is common knowledge that fruit, fresh vegetables and other plant products are healthy and indispensable components of human nutrition. Plant-derived proteins, fibres, vitamins, metal ions etc. have been established as particularly beneficial constituents of food.^45^ The physiological effects of the ubiquitous Chls, on the other hand, have been less well documented. Indeed, chlorophyll and its green degradation products are assumed not to be absorbed in the human intestinal tract; adventitious uptake of Chls even appears to be counteracted, according to recent model studies in mice.^46^

**Figure 3:**
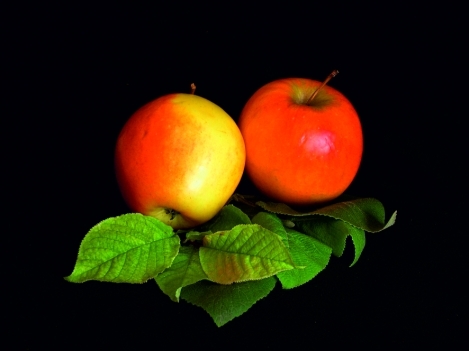
“Ripe” apples.

**Figure 4:**
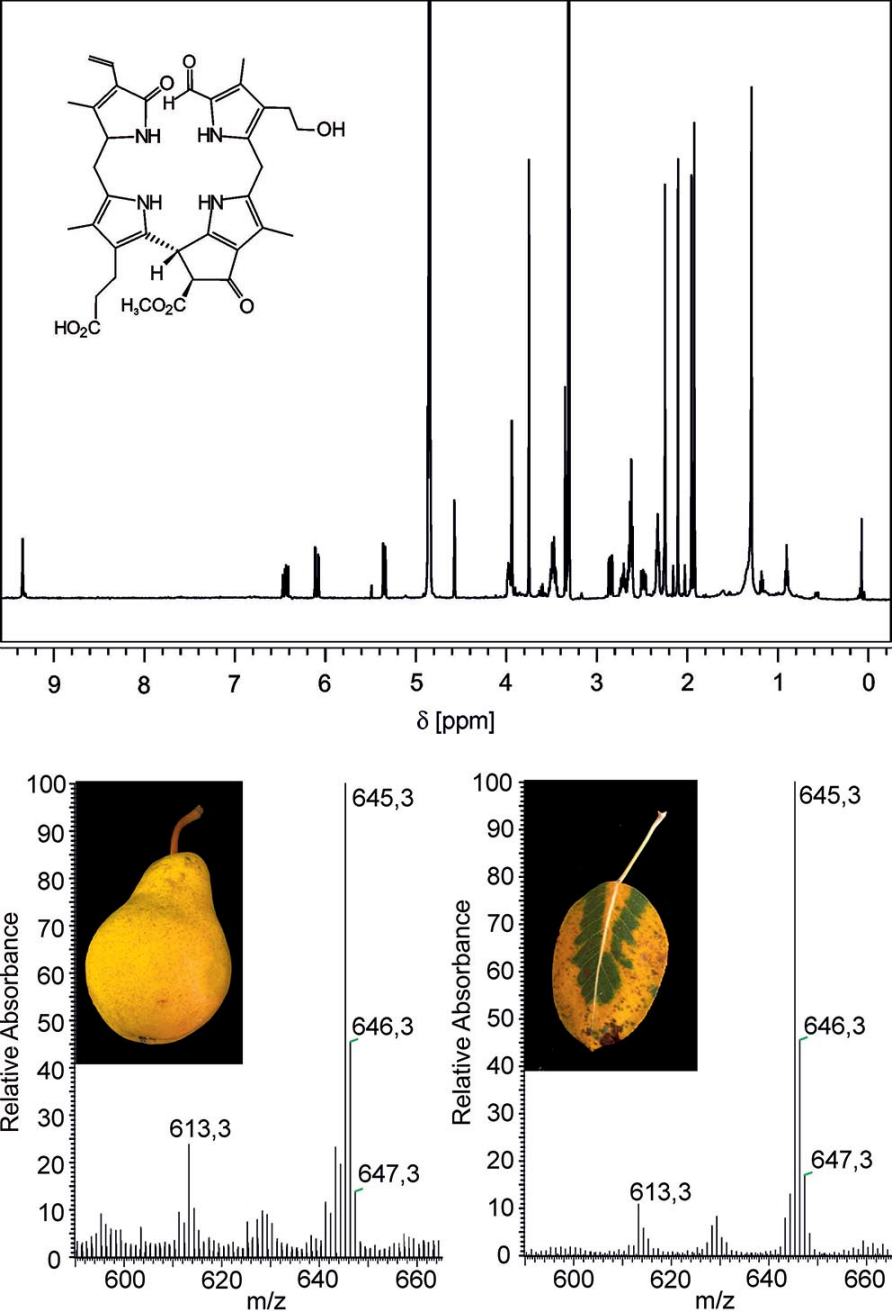
Structure elucidation of “fruit” NCCs by spectroscopic means: 500 MHz ^1^H NMR spectrum (top) and FAB mass spectra of the NCC (*Ms*-NCC-2 = *Pc*-NCC-2 = **2**) from apples andpears.^47^

However, the development of yellow and red colours during ripening of fruit is an indirect sign of breakdown of chlorophyll (see Figure 3), products of which in ripe fruit were unknown until recently.^47^ Analysis of freshly cut and extracted yellow peel of ripe “Golden Delicious” apples (*Malus sylvestris*) and of “Williams” pears (*Pyrus communis*) by high-performance liquid chromatography (HPLC) with detection by UV/Vis spectroscopy revealed the presence of two fractions with UV/Vis characteristics typical of an NCC.^47^ Consistently with related observations on de-greened leaves of senescent plants, the change of colour from green to yellow correlated with increasing amounts of NCCs in the peel of the investigated ripening fruit. Analysis by NMR spectroscopy showed the less polar NCC from apple and pear peels to be a 3^1^,3^2^-didehydro-8^2^-hydroxy-1,4,5,10,15,20,22,24-(21*H*,23*H*)-octahydro-13^2^-(methoxycarbonyl)-4,5-dioxo-4,5-*seco*-phyto-porphyrinate (i.e., to be identical with *Cj*-NCC-1; see above, Table 1 and Figure 4).^47^ By the same means, the more polar fraction was revealed to be a 3^1^,3^2^-didehydro-8^2^-(1-β-glucopyranosyl)-oxy-1,4,5,10,15,20,22,24-(21*H*,23*H*)-octahydro-13^2^-(methoxycarbonyl)-4,5-dioxo-4,5-*seco*-phytoporphyrinate, identical with *Nr*-NCC-2 from tobacco leaves (*Nicotiana rustica*).^15^,^47^ Remarkably, freshly cut and extracted senescent (yellow) leaves of a pear tree both contained the same two NCC fractions.^47^ These findings indicated a substantial similarity of chlorophyll breakdown both in senescent leaves and in ripening fruit and suggested that the colour changes observed in ripening fruit and in senescent leaves were accompanied by a remarkably common biochemical path of chlorophyll breakdown.

**Figure 5:**
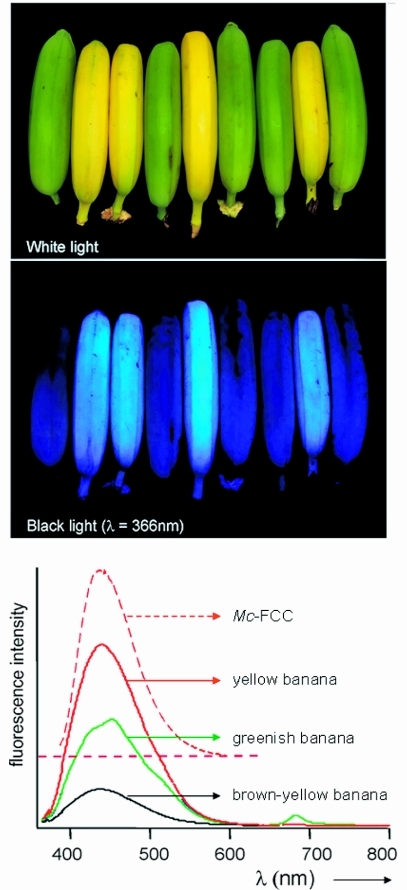
Yellow bananas are blue luminescent. Yellow ripe and green unripe bananas pictured (top) under white light or (middle) UV light at 366 nm and (bottom) luminescence spectra of intact ripened bananas, when greenish (green line), bright yellow (red line) and brown-yellow (black line), and of *Mc*-FCC (in methanol, dashed red line), with excitation at 350 nm.^48^

Surprisingly, in yellow banana peel (*Musa cavendish*) the most abundant chlorophyll catabolites turned out to be polar fluorescent chlorophyll catabolites (FCCs), which accumulated during the ripening process in the peels (see Figure 5).^48^ As described above, FCCs are only fleetingly observed products of chlorophyll breakdown and are believed usually to isomerize to nonfluorescent chlorophyll catabolites in a rapid nonenzymatic process in the acidic vacuoles. Apparently, the situation in the banana is different: in extracts of yellow banana peels, polar FCCs are abundant and occur in great variety. They are easily detectable as some of the most abundant and intensive peaks in an HPLC analysis.

Spectroscopic structural analysis of the most abundant FCC in yellow banana peels – *Mc*-FCC – showed it to have an ester function at the propionate side chain.^48^ The presence of such a modification of the propionic acid side chain was unprecedented in the natural FCCs. However, as described above, it hampers the natural FCC to NCC conversion under slightly acidic conditions and helps to explain the accumulation of *Mc*-FCC in yellow banana peels.

**Figure 6:**
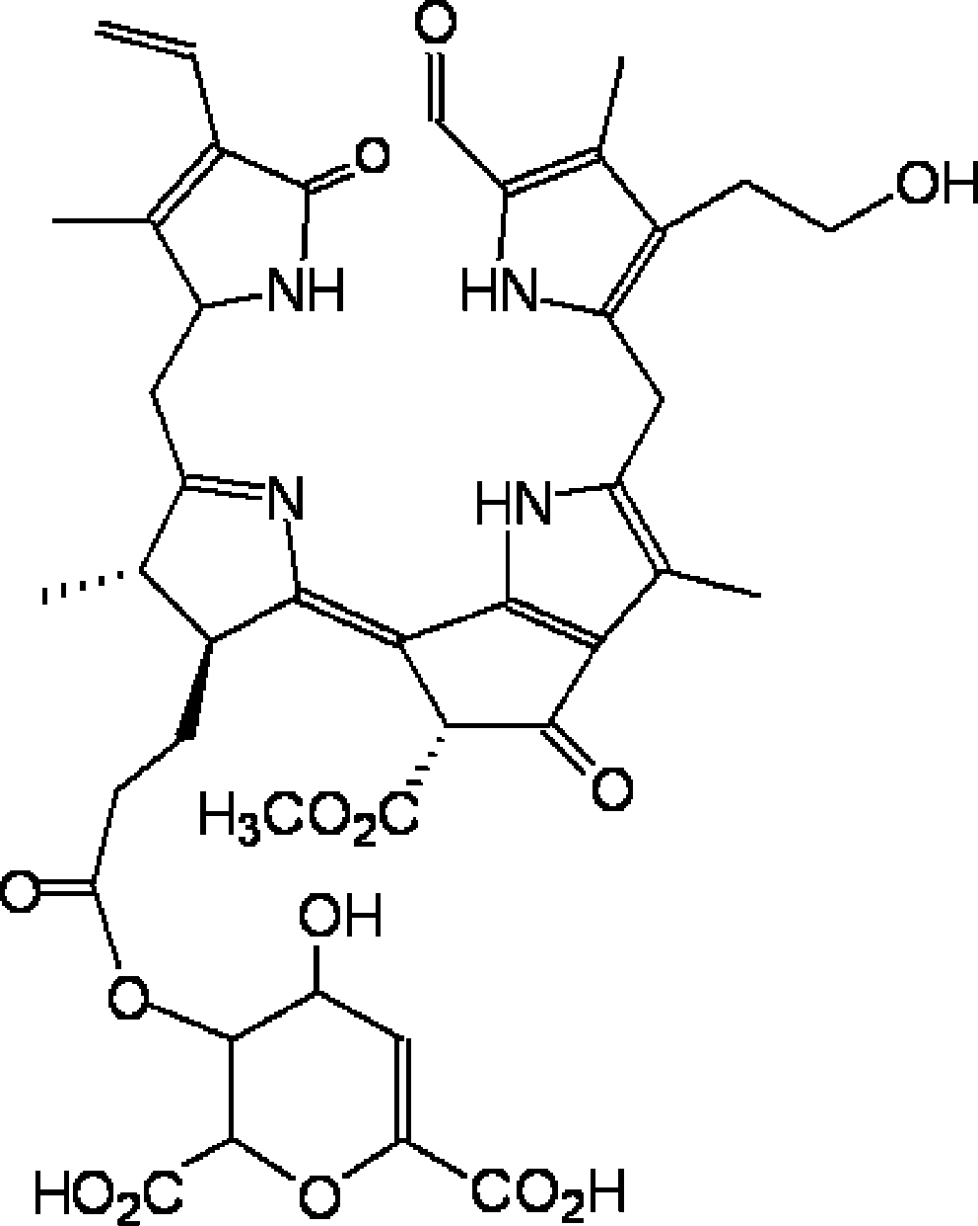
Chemical formula of *Mc*-FCC, the main FCC from banana peel (*Musa cavendish*).^48^

Accumulation of FCCs makes bananas exhibit bright blue luminescence when observed under UV light (see Figure 5). Surprisingly, this luminescence appears to have been overlooked previously. Intact bananas fluoresce with a maximum at 447 nm, the intensity of the fluorescence being highest in fresh ripe, bright yellow bananas, while decreasing again for very ripe bananas that are turning a dull yellow. Extracts of yellow bananas in methanol showed the same fluorescence behaviour as the intact bananas. Likewise, solutions of purified FCCs showed the same fluorescence spectra, as in the case of *Mc*-FCC, the most abundant FCC found in banana peels (see Figures 5 and 6). Clearly, the structure- and mechanism-based stabilization of fluorescent chlorophyll catabolites with respect to isomerization to NCCs and the resulting blue luminescence of bananas is a striking new feature of chlorophyll breakdown.^48^

## Nonfluorescent Chlorophyll Catabolites (NCCs) from Fruit as Antioxidants

**Figure 7:**
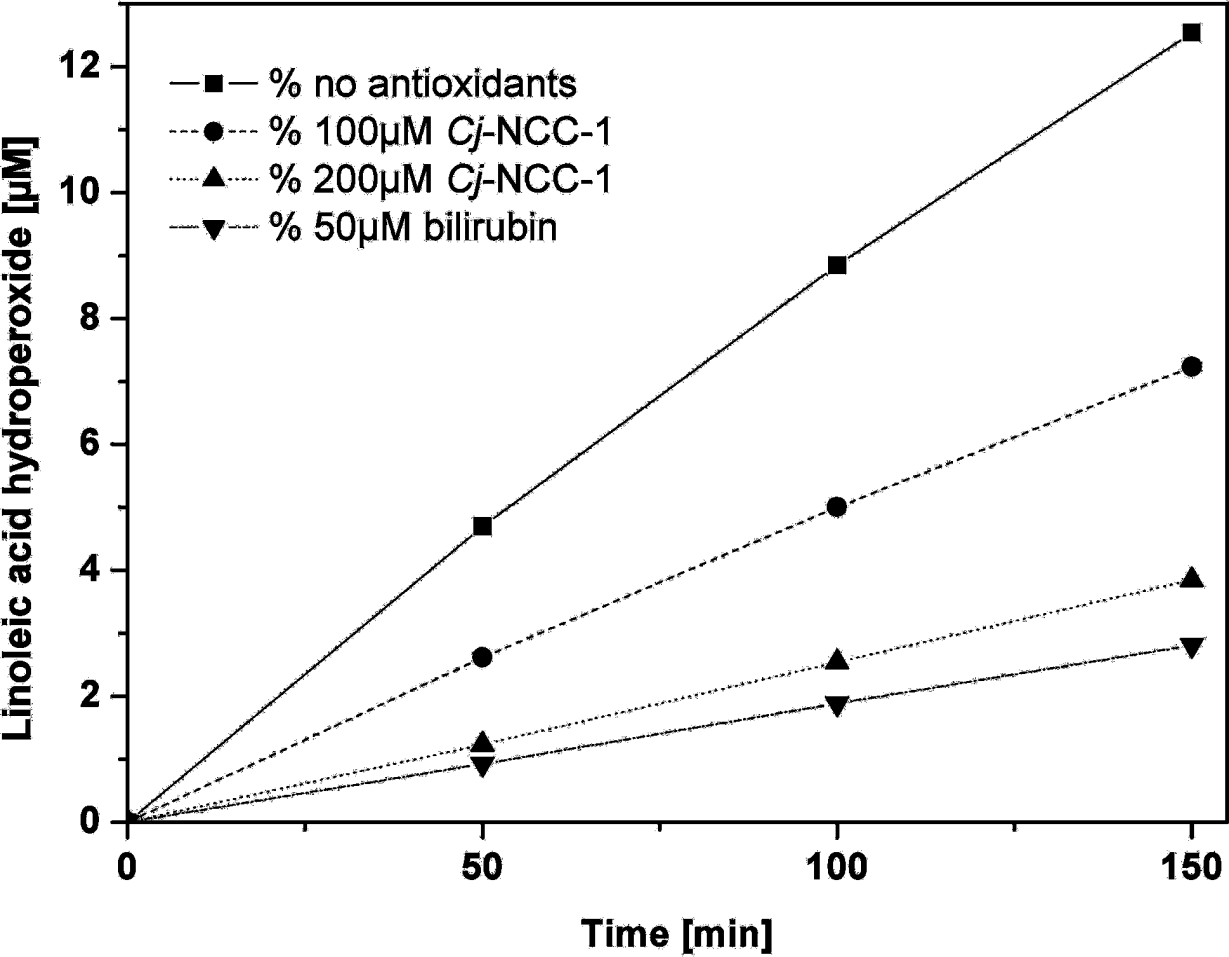
*Cj*-NCC-1 (**2**) as antioxidant. The NCC **2** inhibits the auto-oxidation of linoleic acid.^47^

*Pc*-NCC-2 (= *Cj*-NCC-1 = **2**), the more abundant of the two “fruit” NCCs of intermediate polarity (found in ripening apples and pears),^47^ was also tested in a standard auto-oxidation experiment used for the analysis of bilirubin^49^ (see Figure 7). The rates of formation of hydroperoxides of linoleic acid were monitored (by HPLC analysis) as a function of time and of the concentrations of the added antioxidants. In the presence of the NCC **2**, the rate of formation of hydroperoxides of linoleic acid was significantly reduced. The (concentration-dependent) peroxy radical scavenging effect of **2** was only slightly inferior to that of bilirubin.^49^ These results were of particular interest because nonfluorescent chlorophyll catabolites are structurally related to the tetrapyrrolic heme breakdown product bilirubin and other heme-derived natural linear tetrapyrroles.^43^ Bilirubin has been shown to be an antioxidant^49^ and a cytoprotective component, relevant in reduction of coronary heart diseases, retinal damage and cancer mortality.^50^ The availability of the NCCs in plant-derived nutrition, as documented for apples and pears, calls for their consideration as being of physiological interest in humans (and higher animals) and may give a new twist to the meaning of the old saying “An apple a day keeps the doctor away”.

## Chlorophyll Breakdown and Autumn Colors

**Figure 8:**
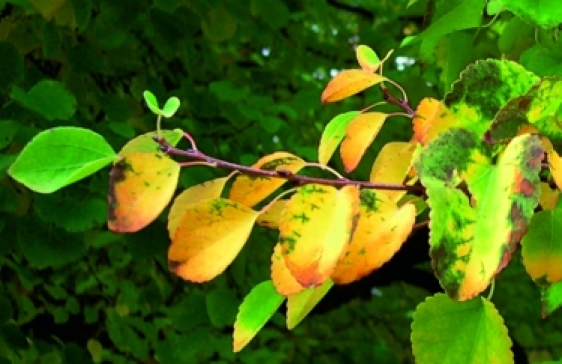
A branch of a Katsura tree (*Cercidiphyllum japonicum*) with yellowing leaves.^52^

**Scheme 6:**
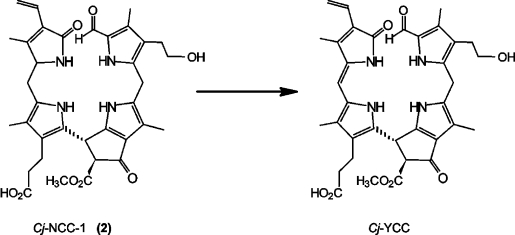
Oxidation of *Cj*-NCC-1 (**2**) with dicyanodichlorobenzoquinone (DDQ) yields *Cj*-YCC, a yellow tetrapyrrolic chlorophyll catabolite.^52^

The appearance of autumn colours is commonly associated with chlorophyll breakdown. In spite of intensivesearch for coloured chlorophyll catabolites in senescent higher plants, such compounds have remained elusive, and NCCs, the colourless and “nonfluorescent” linear tetrapyrroles, have typically been considered to be the rapidly formed “final” tetrapyrrolic products of chlorophyll catabolism in higher plants.^4^ Studies by Losey and Engel revealed the existence of colourless “urobilinogenoidic” chlorophyll catabolites in senescent leaves of barley,^51^ demonstrated to arise from (non-enzymatic?) oxidative deformylation of *Hv*-NCC-1, the main NCC from de-greened primary leaves of barley.^3^ Very recently, we identified a yellow chlorophyll catabolite (YCC) in freshly harvested senescent leaves of a Katsura tree (*Cercidiphyllum japonicum*; see Figure 8).^52^ This natural *Cj*-YCC was characterized by comparison with an oxidation product from *Cj*-NCC-1 (**2**; see Scheme 6), the abundant NCC in *C. japonicum*. The structure of *Cj*-YCC indicated the “western” *meso* position to be unsaturated, opening up conjugation of the two “western” pyrrole rings, and giving rise to a chromophore related to the one found in bilirubin.^53^ These findings represented structural guidelines of how NCCs may be oxidized chemically (when functioning as antioxidants). The isolation of a yellow chlorophyll catabolite from senescent leaves perhaps also points to chlorophyll breakdown as providing direct contributors to autumn colours.^52^ Indeed, a role of NCCs as antioxidants in senescing tissue may also be anticipated (see above). All this provides increasing evidence for further endogenous transformations of chlorophyll catabolites in senescent plants, beyond the stage of the NCCs.

## Conclusions

Chlorophyll breakdown in senescent plants and in ripening fruit provides a natural route to a new group of ubiquitous linear tetrapyrroles, which are distantly related to the heme-derived bilins.^53^,^54^ Further exploration of the structures and of the chemistry of chlorophyll catabolites are of interest and are underway, as are studies on their possible physiological effects in plants and in other organisms that use plant-derived components for their food.
